# IL-22 Promotes IFN-γ-Mediated Immunity against *Histoplasma capsulatum* Infection

**DOI:** 10.3390/biom10060865

**Published:** 2020-06-05

**Authors:** Morgana K.B. Prado, Caroline Fontanari, Camila O.S. Souza, Luiz G. Gardinassi, Karina F. Zoccal, Francisco W.G. de Paula-Silva, Ana P.F. Peti, Carlos A. Sorgi, Alyne F.G. Meirelles, Simone G. Ramos, José C. Alves-Filho, Lúcia H. Faccioli

**Affiliations:** 1 Departamento de Análises Clínicas, Toxicológicas e Bromatológicas da Faculdade de Ciências Farmacêuticas de Ribeirão Preto, Universidade de São Paulo, Ribeirão Preto, São Paulo 14040-903, Brazil; morganaprado@usp.br (M.K.B.P.); fontanari@usp.br (C.F.); camila.oliveirasilva@usp.br (C.O.S.S.); gustavogardinassi@usp.br (L.G.G.); karina_zoccal4@hotmail.com (K.F.Z.); franciscogarcia@forp.usp.br (F.W.G.P.-S.); ana.quimicausp@gmail.com (A.P.F.P.); sorgi@fcfrp.usp.br (C.A.S.); alynefg@fcfrp.usp.br (A.F.G.M.); 2 Programa de Pós-Graduação em Imunologia Básica e Aplicada da Faculdade de Medicina de Ribeirão Preto, Universidade de São Paulo, Ribeirão Preto, São Paulo 14049-900, Brazil; 3 Departamento de Patologia e Medicina Legal da Faculdade de Medicina de Ribeirão Preto, Universidade de São Paulo, Ribeirão Preto, São Paulo 14049-900, Brazil; sgramos@fmrp.usp.br; 4 Departamento de Farmacologia da Faculdade de Medicina de Ribeirão Preto, Universidade de São Paulo, Ribeirão Preto, São Paulo 14049-900, Brazil; jcafilho@usp.br

**Keywords:** Histoplasmosis, CD4^+^ T cells, IFN-γ, IL-22, nitric oxide

## Abstract

*Histoplasma capsulatum* is the agent of histoplasmosis, one of the most frequent mycoses in the world. The infection initiates with fungal spore inhalation, transformation into yeasts in the lungs and establishment of a granulomatous disease, which is characterized by a Th1 response. The production of Th1 signature cytokines, such as IFN-γ, is crucial for yeast clearance from the lungs, and to prevent dissemination. Recently, it was demonstrated that IL-17, a Th17 signature cytokine, is also important for fungal control, particularly in the absence of Th1 response. IL-22 is another cytokine with multiple functions on host response and disease progression. However, little is known about the role of IL-22 during histoplasmosis. In this study, we demonstrated that absence of IL-22 affected the clearance of yeasts from the lungs and increased the spreading to the spleen. In addition, IL-22 deficient mice (*Il22*^−/−^) succumbed to infection, which correlated with reductions in the numbers of CD4^+^ IFN-γ^+^ T cells, reduced IFN-γ levels, and diminished nitric oxide synthase type 2 (NOS2) expression in the lungs. Importantly, treatment with rIFN-γ mitigated the susceptibility of *Il22*^−/−^ mice to *H. capsulatum* infection. These data indicate that IL-22 is crucial for IFN-γ/NO production and resistance to experimental histoplasmosis.

## 1. Introduction

*Histoplasma capsulatum* is a dimorphic pathogenic fungus broadly distributed around the world, with high incidence in Asia, Africa and Americas [1,2]. Histoplasmosis initiates upon inhalation of conidia, the primary infectious form of *H. capsulatum*, producing a spectrum of illnesses that range from acute pulmonary disease to a progressive disseminated form [3]. Disseminated histoplasmosis presents a high morbidity and mortality rate and is most common in immunosuppressed individuals or subjects exposed to a massive inoculum of the fungus [4,5]. In the lungs, conidia transform into yeast forms, where they multiply and establish a granulomatous disease [6]. During the early stages of infection, pattern recognition receptors (PRRs) expressed by macrophages, dendritic cells (DCs) and neutrophils trigger intracellular signaling cascades that initiate antifungal responses such as phagocytosis, generation of reactive oxygen or nitrogen species (ROS or RNS), production of cytokines, chemokines and lipid mediators such as eicosanoids [7,8,9]. These bioactive lipids exhibit important functions during infections, including histoplasmosis [10,11,12,13]. Indeed, leukotriene B_4_ (LTB_4_), is required for effective phagocytosis of yeasts, production of ROS and RNS by macrophages, as well as for CD4^+^ T cell recruitment into the lungs of mice infected with *H. capsulatum* [12,14,15].

CD4^+^ T cells are required for successful anti-histoplasma responses [16,17]. Specifically, Th1 and Th17 cytokines have pro-inflammatory properties that culminate in clearance of the fungus [18,19]. In the absence of Th1 signature cytokines, such as TNF-α, IFN-γ, IL-1β and IL-12, infected mice succumb to infection [18,20,21,22]. IL-12 and IL-1β have been related to the development and expansion of Th1 cells, stimulating IFN-γ production [21,22]. IFN-γ, in turn, inhibits intracellular growth of *H. capsulatum* by inducing murine macrophages to produce nitric oxide (NO), like TNF-α does [20,23,24,25]. In addition, TNF-α antagonism also leads to the generation of T regulatory (Treg) specific T cells (CD4^+^CD25^+^) that inhibit the Th1 immune response [26]. Cytokines of Th17 profile have been related to *H. capsulatum* protection of mice. IL-17A is up-regulated in the acute phase of *H. capsulatum* infection and its neutralization reduced fungal clearance, decreased cell recruitment and increased IL-6 and IL-10 production with no changes in mice survival [19]. On the other hand, the absence of IL-17, in the context of no IFN-γ, increases mice susceptibility to *H. capsulatum* infection [19]. Furthermore, IL-10 and IL-4 are signature cytokines of Tregs and Th2 profiles, related to impaired clearance and suppression of Th1 response [27].

Interleukin-22 emerged as a new cytokine produced by CD4^+^ T cells, (Th22 profile), CD8^+^ T cells, γδ T cells, NKs, ILCs, neutrophils and macrophages, with multiple functions in host response or disease progression [28,29,30,31,32,33]. IL-22 interacts with its receptor (IL-22R) expressed in non-myeloid cells, such as epithelial cells and keratinocytes to maintain homeostasis and tissue repair [34]. During pulmonary *Cryptococcus neoformans* infection, IL-22 mediates the production of antimicrobial peptides (AMPs), such as lipocalin-2, S100A8, S100A9 and Serum amyloid A-3 [35]. Moreover, IL-22 is critical for the clearance of *Aspergillus fumigatus* yeasts by a mechanism that is independent of AMPs [36]. Also, during *Candida albicans* infection, IL-22 regulates the activation of NLRP3 inflammasome by inducing activation of NLRC4 and IL-1ra production, which limits *C. albicans*-induced inflammation [37]. IL-22 is produced in the lungs of mice infected with *H. capsulatum*; however, its contribution to disease progression remains unknown [38,39].

Due the central role of IL-22 in anti-fungal immunity, we sought to explore its function during experimental histoplasmosis. We demonstrated that the absence of IL-22 abrogated the clearance of yeasts from the lungs and increased the spreading to the spleen. Furthermore, *Il22*^−/−^ mice succumbed earlier to infection, which was associated with increased local production of TNF-α and IL-1β, reduced IL-1ra, as well as increased lung inflammation. Seven days after infection, IL-22 deficiency also culminated in a reduction in IFN-γ or IL-17-producing CD4^+^ T cells in the lungs. At a later time point, the IL-22 deficiency leads to accumulation of macrophages (CD45^+^ F4/80^+^ CD11b^+^), neutrophils, (CD45^+^ Ly6C^−^ Ly6G^+^ CD11b^+^) and CD8^+^ T cells. We also observed that *Il22*^−/−^ mice presented reduced transcriptional and protein expression of NOS2 and production of NO_2_^−^ and LTB_4._ Importantly, we found that rIFN-γ, but not LTB_4_ treatment, mitigated the susceptibility of *Il22*^−/−^ mice to *H. capsulatum* infection. Together, our data show that IL-22 is crucial for IFN-γ/NO production and clearance of *H. capsulatum*.

## 2. Materials and Methods

### 2.1. Ethics Statement

This study was carried out in accordance with the recommendations of Conselho Nacional de Controle de Experimentação Animal (“*National Council of the Control on Animal Experimentation*”—CONCEA), the competent authority in Brazil. The protocol was approved on 31 August 2015, by the Ethics Committee on the Use of Animals (CEUA) at Faculdade de Medicina de Ribeirão Preto, Universidade de São Paulo (FMRP/USP), Number 009/2015-1.

### 2.2. Fungal Strain and Culture

The *H. capsulatum* strain used in this study is a clinical isolate from a pulmonary histoplasmosis patient, obtained from Hospital das Clínicas de Ribeirão Preto (Ribeirão Preto, SP, Brazil) [9,11,12,14,15,40,41]. The strain was grown on brain heart infusion (BHI) agar (Difco—Detroit, MI, USA) supplemented with 5% of sterile defibrinated blood sheep (#1189 New Prov, Pinhais PR, Brazil) at 37 °C for 7–10 days. The number of yeasts were determined in Neubauer chamber, adjusted for 1 × 10^7^ mL^−1^ yeasts and submitted for viability assay (Live/Dead *Funga*Light^TM^ Yeast Viability Test—Molecular Probes, Eugene, OR, USA). The yeast cultures were used at ≥95% of viability.

### 2.3. Animals, Treatments and Infection

IL-22 deficient mice (*Il22*^−/−^) and their genetic background C57Bl/6 wild-type (WT), at eight to ten weeks old, males or females age- and sex-matched in all experiments, were obtained from Centro de Criação de Camundongos Especiais (CCCE) of Faculdade de Medicina de Ribeirão Preto (FMRP/USP), Biotério Geral da Prefeitura do Campus USP de Ribeirão Preto (PUSP-RP) and raised at Biosafety Level 3 facility (BL-3) at Laboratório de Vacinas Gênicas da Faculdade de Medicina de Ribeirão Preto (FMRP/USP). Mice were anesthetized by intraperitoneal (i.p.) administration of ketamine and xylazine (75 and 10 mg.kg^−1^ of body weight, respectively,) and infected intratracheally (i.t.) with lethal inoculums (1 × 10^6^ yeasts) of *H. capsulatum* as previously described [11,40].

### 2.4. MiceTtreatments

*Il22*^−/−^ were treated with LTB_4_ (Cayman Chemical Co Ann Arbor, MI, USA) or mouse recombinant IFN-γ (rIFN-γ) (R&D—Minneapolis, MN, USA), per intranasal route (i.n.). LTB_4_ (50 ng/dose) was diluted in sterile PBS 1× and administered twice a day (12/12 h), starting at 24 h before the infection [13]. rIFN-γ (1 µg/dose) was diluted in sterile PBS 1× and administered every day, starting at 4 days after infection [42,43,44]. Control mice received vehicle at the same period as treated mice. Specific antibody treatments with α-Ly6G (Cat: 127620—BioLegend, San Diego, CA, USA) or its isotype control Rat IgG2a, κ (Cat: 400533—BioLegend, San Diego, CA, USA) were used to deplete circulating neutrophils. The antibodies (120 μg/mice) were diluted in sterile PBS 1× and administered at days five and nine post-infection via intraperitoneal route (i.p.). To monitor the efficiency of neutrophil depletion, blood samples of the tail vein were used for differential count using panoptic stain of blood smear.

### 2.5. Survival and Sample Collection

For survival analysis, WT and *Il22*^−/−^ mice, treated or not with LTB_4_ or rIFN-γ or vehicle, were infected with lethal inoculums (1 × 10^6^ yeasts) of *H. capsulatum* and the survival rate was noted every day for 28 days. For sample collection, WT and *Il22*^−/−^ mice were infected with lethal inoculums (1 × 10^6^ yeasts) of *H. capsulatum* and at days two, seven and fourteen post-infection, they were euthanized by cervical dislocation preceded for anesthesia as described above. The lungs and spleen were removed, fractioned and weighted for analyses described below.

### 2.6. Determination of Fungal Burden

Lungs: The left upper lobe was freshly used for fungal burden determination. Tissue was submitted to enzymatic digestion using Liberase (25 µg.mL^−1^—Roche, Mannheim, Germany) and DNase (1 mg.mL^−1^—Sigma Aldrich, St Louis, MO, USA) for 45 min at 37 °C, 150 rpm. Four serial dilutions were inoculated on blood agar and incubated at 37 °C for 3 weeks until yeast growth occurred [11,12,41].

Spleen: The spleen was removed, perforated using a syringe plunger in 1 mL of PBS 1×, plated on blood agar and incubated at 37 °C for 3 weeks until yeast growth occurred [11,12,41].

### 2.7. Flow Cytometry

The same cellular suspension used for fungal burden determination in lungs was used for the immunophenotyping of lung parenchyma cells by flow cytometry, as previous described [45,46]. The cell suspension was filtered in a sterile filcon syringe type (BD Bioscience—San Jose, CA, USA), submitted to red blood cells lysis with ammonium-chloride-potassium (ACK) solution and the number of total cells isolated from lung parenchyma was determined in a Neubauer chamber. The cells were divided for intracellular and extracellular staining. Cells used for intracellular markers were stimulated for 2 h with PMA (50 ng.mL^−1^—Sigma Aldrich), ionomicyn (0.5 µg.mL^−1^—Sigma Aldrich) and brefeldin A (10 µg.mL^−1^—Sigma Aldrich) in RPMI 1640 (Gibco, Itapevi, SP, Brazil) supplemented with 5% of FBS (#10270106 Gibco^®^ Thermo Fisher Scientific—South America, Brazil), 1% of non-essential amino acids (Sigma—St. Louis, MO, USA), 1% gentamicin (Gibco—Grand Island, NY, USA), 1% L-glutamin—200 nM (Sigma, St Louis, MO, USA), 1% Piruvate—200 nM (Sigma—St Louis, MO, USA) and 0.1% of β-mercapto ethanol (Sigma, St Louis, MO, USA). In specific experiments, the cells were stimulated either with 50 ng.mL^−1^ of rIL-18 for 12 h. In the next step, all cells, stimulated or not, were centrifuged (7096× *g*, 1 min at 4 °C) and fixed with PBS 1× 4% formaldehyde. Eleven minutes later, 1 mL of PBS 1× was added in cell suspension and stored at 4 °C overnight. Further, cells used for intracellular stain were washed and incubated with permeabilization buffer (0.1% of sodium azide, 0.2% of saponin, 1% of FBS diluted in PBS 1×) and cells used for extracellular staining were washed with PBS 1× + 2% FBS. The cells were washed twice with permeabilization buffer, centrifuged (7096× *g*, 1 min at 4 °C) and the pellet suspended in 25 µL of FcBlock. Cell suspension was incubated with permeabilization buffer for 20 min at 4 °C and then with mix of antibodies for 30 min at 4 °C. The following antibodies were used: CD4 (clone: RM4-5); CD3 (clone: 17A2); CD8a (clone: 53-6.7); IL-17A (clone: Tc11-18H10); IL-10 (clone: JES5-16E3) and CD45 (clone: 30-F11) were purchased from BD Biosciences (BD Bioscience, San Jose, CA, USA); IFN-γ (clone: XMG1.2); F4/80 (clone: BM8); CD11b (clone: M1/70); Ly-6C (clone: HK1.4) and Ly-6G (clone: RB6-8C5), were purchased from eBioscience (Affymetrix, Carlsbad, CA, USA). After the period of incubation, the cells were washed with permeabilization buffer and PBS 1× and finally suspended in Cytofix Fixation Buffer (554655, BD Bioscience—San Diego, CA, USA). Data were acquired in FACSCanto I, FACSCanto II and LSRFortessa cytometers (BD Bioscience, San Jose, CA, USA) and analyzed with FlowJo software (Ashland, OR, USA). The gates were defined using the “Anti-Hamster Ig κ / Negative Control Compensation Particles Set” (# 552845 BD Bioscience, San Jose, CA, USA), as well as unstained and single-stained cell controls.

### 2.8. Histopathological Analysis

The right middle lobe from all samples was used for histological analysis. The lobes were removed, fixed with 10% buffered formalin and embedded in paraffin. Two to three random cuts of 5 µm of all animals were stained with hematoxilin and eosin (HE) for analysis of cellular content, or with Grocott-Gomori’s methenamine silver stain (GMS), for the determination of fungal load in lung parenchyma [13,46]. Lung sections were also used for quantification of NOS type 2 enzyme by immunohistochemistry. The tissues were immersed in 3% hydrogen peroxide for 10 min to block endogenous peroxidase activity, incubated in normal horse serum (ab7484 Abcam—North America origin) to block non-specific binding and then incubated with anti-NOS-2 antibody (#160862—Cayman Chemical Co Ann Arbor, MI, USA). The lungs were washed, incubated with anti-IgG biotinilated (Vector Laboratories, Burlingame, CA, USA) for 1 h at room temperature and with complex avidin-biotin-peroxidase (Vector Laboratories, Burlingame, CA, USA). The reaction was revealed by the addition of 3, 3-diaminobenzidine tetrahydrochloride (DAB, Vector Laboratories, Burlingame, CA, USA) and incubation with Mayer’s hematoxylin. Five to ten random photomicrograph sections (400x resolution) were obtained with optical microscope Zeiss (Axio Scope.A1—Dublin, CA, USA) from all mice strains, and following all criteria for proper camera setup. The images were converted to gray-scale (RGB 8-bit image) and the particles were quantified using ImageJ software (U.S. NIH, Bethesda, MD, USA) to estimate the percentage of lung area covered by infiltrating cells or covered by yeasts. These methods for quantify lung inflammation and fungal load were applied as previously described [13,41,46].

### 2.9. Quantification of Cytokines by ELISA and NO_2_^−^ by Griess Reaction

The right upper and lower lobes were processed for protein, cytokine and lipid mediator quantification. The lungs were homogenized using Turrax (IKA, Labortechnik, Staufen, Germany) with 1 mL of RPMI-I for each 100 mg of tissue. Then, the samples were centrifuged (753× *g* 10 min, 4 °C) and the supernatant removed and fractioned for lipid mediators or for cytokines quantification [41]. For lipid mediators, 500 µL of supernatants was mixed with methanol (*v/v*) and stored at -80 °C until lipid extraction. For cytokines quantification, the supernatant of the samples were mixed with protease inhibitor at 1.6 mM (Complete, Roche, Indianapolis, IN, USA) and stored at −80 °C, at most for four months in aliquots of 250 μL and avoiding freeze and thawing, until quantification of cytokines. ELISA assay was performed for IFN-γ, IL-17, IL-22, TNF-α, IL-1β, IL-1ra, IL-12p70 and IL-23 (R&D Systems, Minneapolis, MN, USA) and IL-18 (MBL International Corporation, Woburn, MA, USA) according to the manufacturer’s instructions. NO_2_^−^ concentration (μM) was measured in lung homogenates using the Griess solution. Serial dilutions with NaNO_2_ (Sigma Aldrich, St. Louis, MO, USA) were used standard curves in order to estimate NO levels as previously described [14].

### 2.10. Quantification of LTB_4_ by HPLC/MS/MS

To the homogenate supernatant fractioned for quantification of LTB_4_, a solution with internal deuterated standards (Cayman Chemical Co Ann Arbor, MI, USA) was added in a known concentration for the identification of LTB_4_. Samples were acidified with 50 µL of HCl (1N), centrifuged and applied in HYPERSEP C18 column (Thermo Scientific, Rockwood, TN, USA) as previously described [41]. The HPLC/MS/MS (LC Shimadzu—ESI Triple TOF 5600+ SCIEX) method for identification and quantification of lipid mediators was optimized using stationary phase chromatographic column C18 Ascentis EXPRESS (Supelco—St. Louis, MO, USA) [47]. The data processing was obtained by the PeakView 2.1 and MultiQuant 3.0 programs (AB Sciex, Foster, CA, USA).

### 2.11. qRT PCR

The lower left lobe was used for gene expression analyses. Total RNA was extracted using guanidine-based columns (Purelink, Ambion Invitrogen, Carlsbad, CA, USA) and quantified by fluorometric method (Qbit, Invitrogen, Carlsbad, CA, USA). Complimentary DNA (cDNA) was synthesized from 1.5 μg of total RNA using commercially available reverse transcriptase kits (High Quality cDNA Reverse Transcriptase Kit, Applied Biosystems, Carlsbad, CA, USA). Fifty nanograms (2 μL) of total cDNA was amplified by quantitative reverse transcriptase-polymerase chain reaction (qRT–PCR) using TaqMan primers for *Nos2* (Mm02524428_g1), in a StepOne Plus machine (Applied Biosystems, Foster City, CA, USA). *Gapdh* (Mm99999915_g1) was used as a reference gene and to normalize expression levels by the ^ΔΔ^Ct method. The expression data were presented as n-fold difference relative to the control group [13].

### 2.12. Statistical Analysis

Mean values were compared between groups by a one-way ANOVA followed by the Newman–Keuls multiple comparison test, or by two-way ANOVA followed by Bonferroni’s multiple comparison test or by Student’s *t*-test. Survival differences between groups were calculated using a log-rank test. Analyses were performed using the Prism 5 software (Graphpad Prism, La Jolla, CA, USA). A *p* < 0.05 was considered significant.

## 3. Results

### 3.1. IL-22 Is Critical for Host Resistance to H. capsulatum Infection

**Figure 1:**
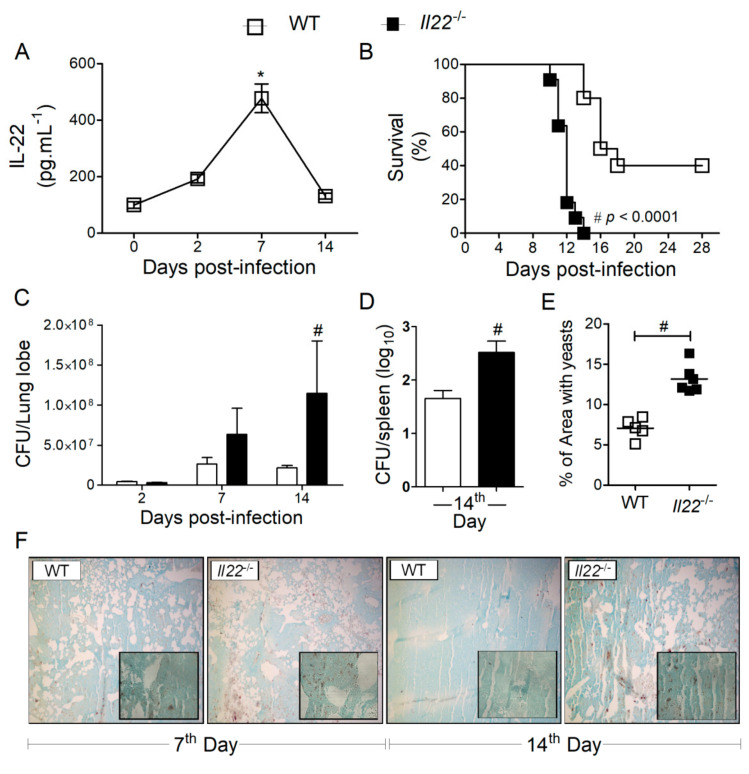
The absence of IL-22 increases the fungal load and the susceptibility of mice infected with *Histoplasma capsulatum*. Eight-week-old C57Bl/6 mice (WT) were infected i.t. with a lethal inoculum (1 × 10^6^ yeasts) of *H. capsulatum*. On days 2, 7 and 14 post-infection, the mice were euthanized, the lungs removed and homogenized for quantification of IL-22 by ELISA (**A**). WT or *Il22*^−/−^ mice were infected i.t. with a lethal inoculum (1 × 10^6^ yeast) of *H. capsulatum* and observed daily for determination of the survival curve (**B**). Data were analyzed by log-rank test and Mantel–Cox in the post-test (*n* = 10). In independent experiments, infected mice were euthanized on days 2, 7, and 14 post-infection, and the lungs (**C**) and spleens (**D**) were removed and processed for fungal load analysis by yeast colony forming units (CFU) assay. Data are expressed as mean ± SEM of three independent experiments (*n* = 6–11) and analyzed by the two-way ANOVA test and Bonferroni in the post-test or analyzed by Student’s *t*-test (*n* = 5). Data were considered statistically significant when *p* < 0.05, * infected vs. uninfected and # WT + *Hc* vs. *Il22*^−/−^ + *Hc*. The mean right lobe was removed on days 7 and 14 after infection, fixed in formalin and embedded in paraffin. Five-micrometer sections were stained by the Grocott’s method through impregnation of silver methenamine (GMS), and analyzed by light microscopy, by 100× and 400× (in set) resolution (**F**). Images obtained on 7th day post-infection were quantified in the ImageJ software, regarding % of area covered by yeasts (**E**). Data are expressed as mean ± SEM of a representative experiment (*n* = 4–6) and analyzed by Student’s *t*-test.

Previous investigations have shown that mice infected with both forms of *H. capsulatum*, conidia or yeasts, produce IL-22 in the lungs. Using mycelial propagules (conidia and hyphal fragments), the production of IL-22 was increased at 12 h, 7 and 28 days post-infection in Balb/c mice [38]. Moreover, using 2 × 10^6^ yeasts, IL-22 production was increased at the 7th day of infection in C57Bl/6 mice [39]. We confirmed that IL-22 is produced and augmented at the 7th day of infection in the lungs of C57Bl/6 mice, which were infected with 1 × 10^6^ yeasts of a different *H. capsulatum* strain (Figure 1A). To understand the role of IL-22 during experimental histoplasmosis, we monitored the mortality rate of C57Bl/6 (WT) or *Il22*^−/−^ mice infected with *H. capsulatum* for 28 days. Strikingly, 100% of *Il22*^−/−^ mice died 15 days after infection, contrasting with 40% of survival of WT animals at the end of 28 days (Figure 1B). In addition, we evaluated the fungal burden in the lungs and spleens after 2, 7 and 14 days post-infection. The absence of IL-22 impaired the clearance of the yeasts in the lungs (Figure 1C) and promoted spreading to the spleen at the 14th day post-infection (Figure 1D). We confirmed increased *H. capsulatum* load in the lungs of *Il22*^−/−^ mice by staining fungal cells with Grocott’s methylamine silver stain (Figure 1F) and measuring the area of the lungs covered by yeasts using ImageJ at the 7th day post-infection (Figure 1E).

### 3.2. IL-22 Regulates Lung Inflammation during H. capsulatum Infection

**Figure 2:**
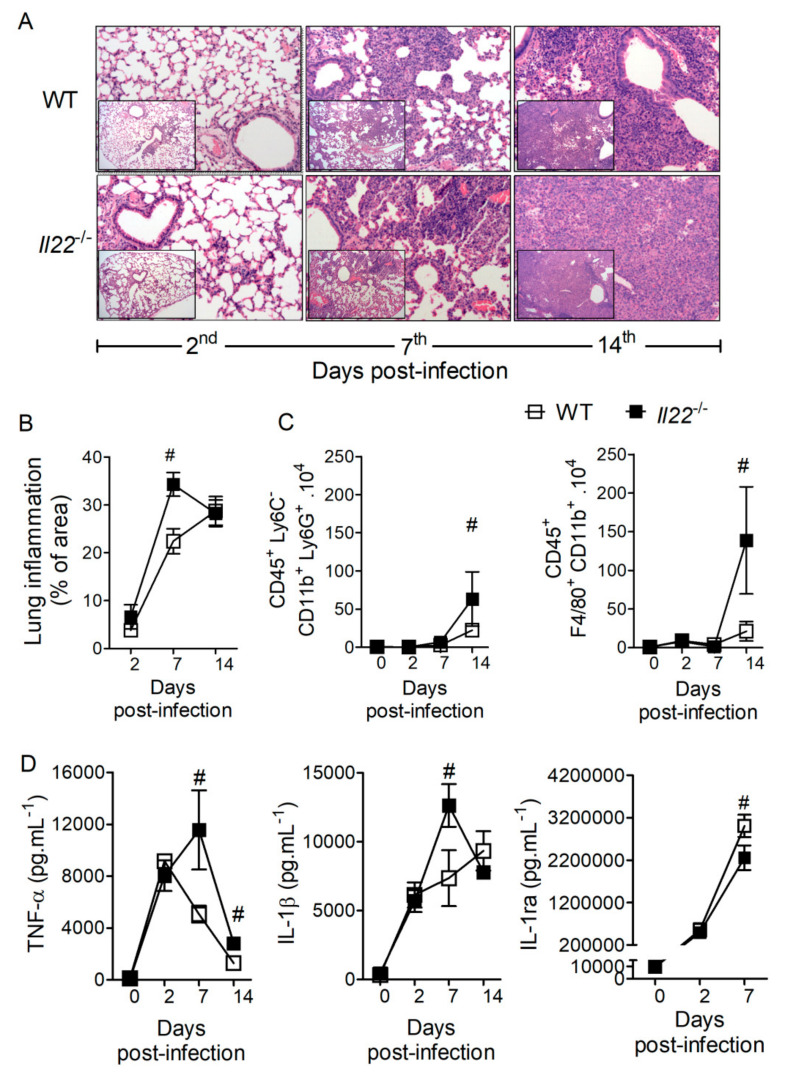
IL-22 deficiency enhances the recruitment of neutrophils and macrophages and modulates the cytokines IL-1β, TNF-α and IL-1ra in *H. capsulatum* infected lungs. Eight-week-old WT or *Il22*^−/−^ mice were infected i.t. with a lethal inoculum (1 × 10^6^ yeasts) of *H. capsulatum* and euthanized on days 2, 7 and 14 after infection. The mean right lobe was removed, fixed in formalin and embedded in paraffin. Five-micrometer sections were stained by HE and analyzed by light microscopy, with magnification of 200× and 100× (in set) (**A**). Images were quantified in the ImageJ software, regarding % of infiltrated area (**B**). On days 2, 7 and 14 after infection, the upper left lobe was removed, disrupted, counted and evaluated by flow cytometry for the presence of neutrophils CD45^+^ Ly6C^−^ Ly6G^+^ CD11b^+^ and macrophages CD45^+^ F4/80^+^ CD11b^+^ (**C**). The absolute number of cells is shown as mean ± SEM, of two independent experiments (*n* = 4–16). To determine IL-1β, TNF-α and IL-1ra production, the lower and upper right lobes were removed, homogenized and the supernatants used for quantification by ELISA (**D**). Data are expressed as mean ± SEM of two independent experiment (*n* = 4–16) and analyzed by the two-way ANOVA and Bonferroni test in the post-test. Data were considered statistically significant when *p* < 0.05, # WT + *Hc* vs. *Il22*^−/−^ + *Hc*.

In view of the high susceptibility of *Il22*^−/−^ mice to *H. capsulatum* infection, we sought to determine immunopathological features associated with IL-22 deficiency. Histological analyses of the lungs at 2, 7 and 14 days post-infection demonstrate that mice from both genotypes exhibited increased cellular infiltration over the course of infection (Figure 2A), but *Il22*^−/−^ animals presented greater lung inflammation 7 days after infection with *H. capsulatum* (Figure 2B). Flow cytometric analysis of lungs on the same time points of infection revealed a time-dependence on the accumulation of neutrophils (CD45^+^, Ly6C^−^, Ly6G^+^ and CD11b^+^) and macrophages (CD45^+^, F4/80^+^ and CD11b^+^) in both genotypes (Figure 2C). Furthermore, we observed that IL-22 deficiency promoted increased accumulation of neutrophils and macrophages 14 days after infection (Figure 2C). Neutrophils and macrophages are important sources of pro- and anti-inflammatory mediators such as cytokines. Therefore, we determined the production of TNF-α, IL-1β and IL-1ra in the lungs after infection. Compared to WT animals, both TNF-α and IL-1β increased in the lungs of *Il22*^−/−^ mice at the 7th day of infection (Figure 2D), correlating with increased lung inflammation at the same time point. Interestingly, IL-1ra, an anti-inflammatory cytokine, was reduced in infected IL-22-deficient mice when compared to WT (Figure 2D). In addition, we observed that IL-22 deficiency resulted in increased gene expression of diverse innate immune molecules in the lungs after infection, including *Itgb2, Tlr2, Clec7a, Cysltr1*, *Cd36* and *Ltb4r1* after 7 days, and *Tlr2, Cd36, Ltb4r1, Alox5ap, Cd14, Tlr4, Ticam2, Mrc1, Myd88, Tlr6, Alox5, Ptgs2, Ptgse2, Tlr1* and *Ticam1* after 14 days of infection (Figure S1). Once IL-22 deficiency induced exacerbated neutrophil recruitment to the infected lungs, we checked whether neutrophil accumulation was involved in the mortality of the animals. So, to deplete circulating neutrophils and to prevent neutrophil accumulation in the lungs, *Il22*^−/−^ mice were treated with anti-Ly6G (120 μg i.p.), or its isotype control as described (Figure S3). Anti-ly6G treatment was effective in depleting circulating neutrophils in IL-22 deficient mice; however, preventing neutrophil accumulation in the lungs did not reduce mice mortality during *H. capsulatum* infection (Figure S3). Collectively, these data suggest that IL-22 is important to regulate the recruitment and activation of innate immune cells during *H. capsulatum* infection. However, the intense neutrophil recruitment, per se, is not responsible for *Il22*^−/−^ mice mortality.

### 3.3. IL-22 Operates Independently of LTB_4_ and AMPs to Protect against H. capsulatum Infection

**Figure 3:**
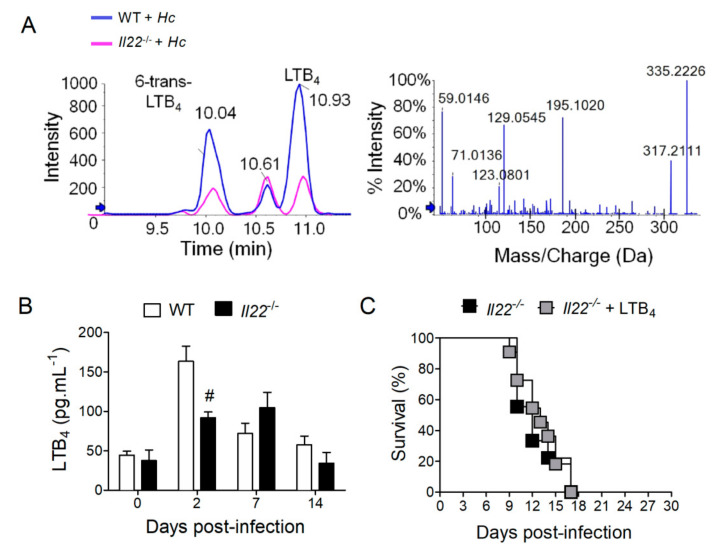
IL-22 absence compromised the production of LTB_4_ in infected lungs, but LTB_4_ treatment did not rescue *Il22*^−/−^ mice from death. Eight-week-old WT or *Il22*^−/−^ mice were infected i.t. with lethal inoculum of *H. capsulatum* and euthanized on days 2, 7 and 14 after infection. The lower and upper right lobes were removed, homogenized and the supernatant was used for lipid extraction in C18 column for quantification of LTB_4_ by HPLC/MS/MS (**B**). Representative samples showing the chromatographic peaks (**A**—left panel) and mass fragmentation spectrum (**A**—right panel) of LTB_4_ on 2^nd^ day post-infection. Values were expressed as mean ± SEM of two independent experiments (*n* = 4–7) and analyzed by the two-way ANOVA test and Bonferroni test in the post-test. Ten-week-old *Il22*^−/−^mice infected with lethal inoculum of *H. capsulatum* were treated 2× a day (in) with 50 ng LTB_4_, starting 24 h before infection. The animals were observed daily to determine the survival curve (**C**). Data were analyzed by log-rank test and Mantel–Cox in the post-test (*n* = 10). Data were considered statistically significant when *p* < 0.05, #WT + *Hc* vs. *Il22*^−/−^ + *Hc* or #*Il22*^−/−^ + *Hc* vs. *Il22*^−/−^ + *Hc* + LTB_4._

LTB_4_ is a potent bioactive lipid that performs important functions during histoplasmosis [12,14,15]. Thus, we hypothesized that IL-22 deficiency could impair LTB_4_ production during infection. To test this hypothesis, WT and *Il22*^−/−^ mice were infected with *H. capsulatum* and lungs were collected at designated time points for the analysis of LTB_4_ production by LC-MS/MS. Chromatographic peaks (Figure 3A—left panel) and mass fragmentation (Figure 3A—right panel) demonstrated the identification of LTB_4_ and other lipids in the lungs of infected animals. Further analysis revealed that lungs from *Il22*^−/−^ mice infected with *H. capsulatum* only presented significantly lower concentrations of LTB_4_ on the 2nd day after infection (Figure 3B). This suggests that failure to produce adequate amounts of LTB_4_ early after infection is the cause of the higher susceptibility of *Il22*^−/−^ mice to *H. capsulatum*. Therefore, *Il22*^−/−^ mice were treated with LTB_4_ (50 ng) or vehicle one day before, and during infection with *H. capsulatum*, while survival was monitored for 28 days. Surprisingly, we found that LTB_4_ treatment was unable to inhibit animal mortality, which were as susceptible as mice that received vehicle (Figure 3C). This indicates that although IL-22 is necessary for maximal LTB_4_ production, this cytokine coordinates other molecular mechanisms that mediate protection against *H. capsulatum* infection.

IL-22 is known to induce the secretion of AMPs in the skin and intestine, which promotes pathogen killing, and could thus mediate resistance to *H. capsulatum* infection [48,49]. We observed a significant reduction in the gene expression of α-defensin-1 (*Defa1*) in the lungs of *Il22*^−/−^ mice after 14 days of infection (Figure S2). However, *Defa1* expression in WT mice was similar to that of non-infected animals after 14 days of infection, suggesting that this AMP does not play a significant role during *H. capsulatum* infection. In addition, the gene expression of lipocalin-2 (*Lcn2*), β-defensin-1 (*Defb1*) or even mucin 5 subtypes a and c (*Muc5ac*) were similar between both genotypes (Figure S2). Cathelicidin antimicrobial peptide (*Camp*), on the other hand, was upregulated on the 14th day post-infection, possibley due to the increased fungal burden observed at this time-point in *Il22*^−/−^ mice. These data suggest that IL-22 does not coordinate the production of AMPs in the lungs of subjects infected with *H. capsulatum*.

### 3.4. IL-22 Deficiency Impacts T Lymphocyte Responses against H. capsulatum

**Figure 4:**
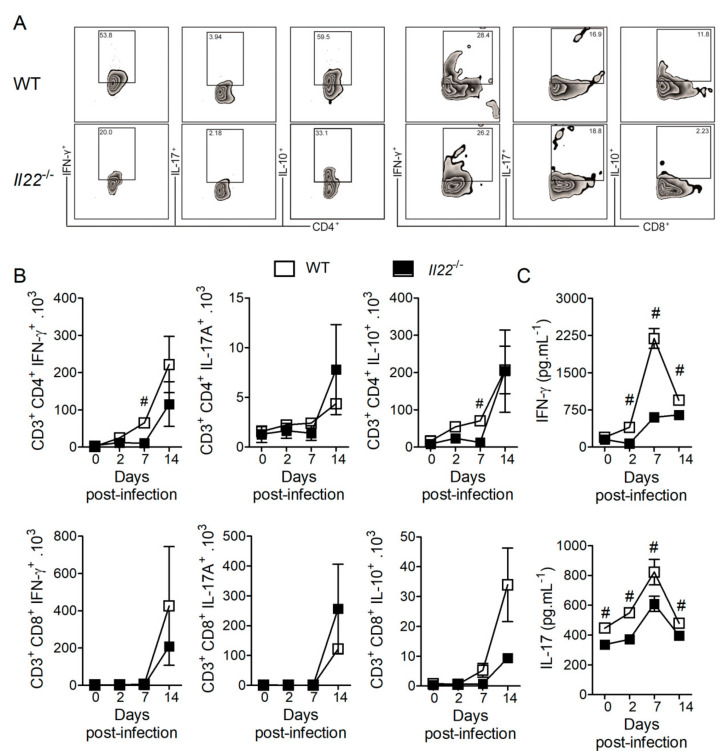
IL-22 deficiency alters frequency of T cells, the production of IFN-γ and IL-17 in *H. capsulatum* infected-lungs. Eight-week-old WT or *Il22*^−/−^ mice were infected i.t. with lethal inoculum (1 × 10^6^ yeasts) of *H. capsulatum* and euthanized on days 2, 7 and 14 after infection. The upper left lobe was removed, disrupted and evaluated by flow cytometry for the presence of Th1 (CD3^+^CD4^+^IFN-γ^+^), Th17 (CD3^+^CD4^+^IL-17A^+^), Treg (CD3^+^CD4^+^IL-10^+^), Tc1 (CD3^+^CD8^+^IFN-γ^+^), Tc17 (CD3^+^CD8^+^IL-17A^+^) and TcRegs (CD3^+^CD8^+^IL-10^+^). Representative zebra plots of CD4 T cells (7 dpi) and of CD8 T cells (14 dpi) were illustrated (**A**). The absolute number was expressed as mean ± SEM, of two independent experiments (**B**). To quantify IFN-γ and IL-17, the lower and upper right lobes were removed, homogenized and the supernatants used for ELISA assay (**C**) (*n* = 4–6). Data were analyzed by the One-Way ANOVA with Newman-Keuls multiple comparison test and considered significant when *p* <0.05, #WT + *Hc* vs. *Il22*^−/−^ + *Hc*.

Effective adaptive immune response against *H. capsulatum* is mediated mainly by Th1 and Th17 lymphocytes, which are characterized by the production of IFN-γ and IL-17, respectively [24,25]. In contrast, Treg cells produce the anti-inflammatory cytokine IL-10, whose absence is associated with protection against *H. capsulatum* infection [50]. To investigate whether IL-22 deficiency affects T cell mediated immunity, we performed flow cytometry of cellular suspensions obtained from lungs of WT and *Il22*^−/−^ mice over the course of infection with *H. capsulatum.* The gating hierarchy used to identify lymphocytes is illustrated in Figure S4 and one representative plot of percentages of T cell subsets is shown in Figure 4A. In the absence of IL-22, we observed reduced numbers of IFN-γ- or IL-10-producing CD4^+^ T cells, but not IL-17-producing CD4^+^ T cells at seven days post-infection (Figure 4A—left panel and 4B—upper panels). Interestingly, we observed reduced concentrations of both IFN-γ and IL-17 along the course of the infection, although biologically significant differences were identified only at the seventh day post-infection (Figure 4C). This suggests that CD4^+^ T cells are the main sources of IFN-γ under the instructions of IL-22, while other cells contribute to the production of IL-17. Although we observed changes in the numbers of CD8^+^IFN-γ^+^ or CD8^+^ IL-17A^+^ T cells at 14 days post-infection, they did not reach statistical significance and do not seem to contribute to IL-22-dependent IL-17 production (Figure 4A—right panel and 4B—lower panels).

### 3.5. IL-22 Deficiency Impacts Nitric Oxide (NO) Production in the Lungs of H. capsulatum-Infected mMice

**Figure 5:**
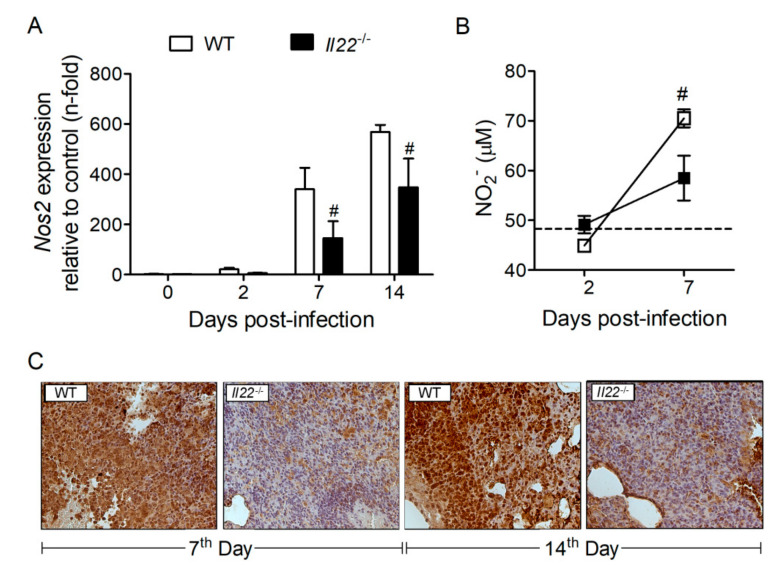
NOS type 2 and NO_2_^−^ were diminished in lung parenchyma of *Il22*^−/−^ mice. Eight-week-old WT or *Il22*^−/−^ mice were infected i.t. with lethal inoculum (1 × 10^6^ yeast) of *H. capsulatum*. The left lower lobe was removed, and the RNA extracted for analysis of the gene expression of the enzyme Nos2 by RT qPCR (**A**). On days 2 and 7 post-infection, the lower and upper right lobes were removed, homogenized in 1 mL of PBS 1× and the supernatant was used to quantify NO_2_^−^ (μM) by using a Griess reaction (**B**). On days 7 and 14 post-infection the medium right lobe was removed, fixed in formalin and embedded in paraffin. Five-micrometer sections were labeled with anti-Nos2 primary antibody, biotinylated anti-IgG secondary antibody and substrate. The sections were counterstained by Mayer’s hematoxylin and analyzed by light microscopy, at 400× (**C**). Data were expressed as mean ± SEM of a representative experiment (*n* = 4–7) and analyzed by the two-way ANOVA test and Bonferroni post-test and considered significant when *p* < 0.05, #WT + *Hc* vs. *Il22*^−/−^ + *Hc*.

Previous studies showed that IFN-γ induces NOS2 expression and NO production to control *H. capsulatum* infection [23,51]. Considering that IFN-γ was reduced in the lungs of *Il22*^−/−^ mice during *H. capsulatum* infection, we investigated the effect of IL-22 deficiency on transcriptional and protein expression of NOS2. Compared to WT animals, lungs of *Il22*^−/−^ mice exhibited reduced gene and protein expression of NOS2 at 7 and 14 days after infection (Figure 5A, C, respectively). Furthermore, the production of NO was measured indirectly through the quantification of nitrite (NO_2_^−^) in the lung homogenates. Interestingly, in accordance with the expression of NOS2, *Il22*^−/−^ mice exhibited reduced NO production in the lungs, when compared to WT infected mice (Figure 5B).

### 3.6. IFN-γ Treatment Rescues Survival of Il22^−/−^ Mice during H. capsulatum Infection

**Figure 6:**
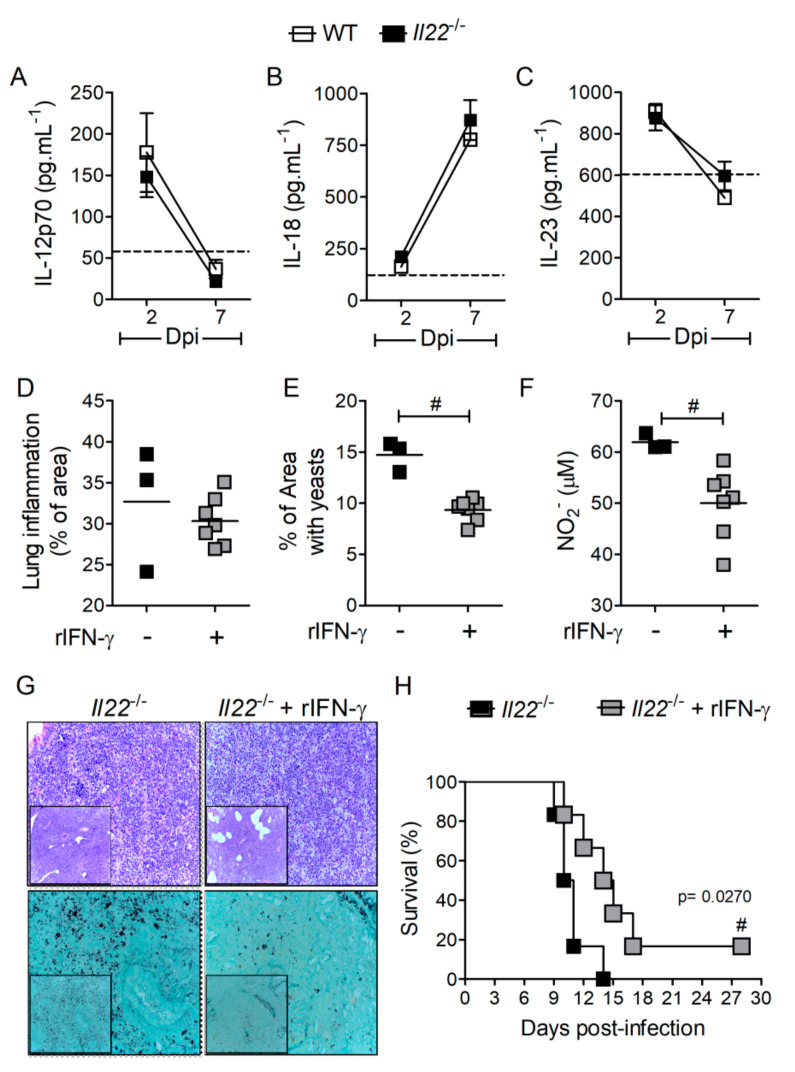
IL-22 absence did not compromise IL-12p70, IL-18 and IL-23 cytokine production in *Hc*-infected lungs, but rIFN-γ treatment, rescued IL-22 deficient mice from death. Eight-week-old WT or *Il22*^−/−^ mice were infected i.t. with lethal inoculum of *H. capsulatum* and euthanized on days 2 and 7 after infection. The lower and upper right lobes were removed, homogenized and the supernatant was used for quantification of IL-12p70 (**A**), IL-18 (**B**) and IL-23 (**C**) by ELISA and NO_2_^−^ (μM) by using a Griess reaction at 7th day post-infection (**F**). (*n* = 4–6). Data were analyzed by the two-way ANOVA test and Bonferroni post-test or by Student’s *t*-test and considered significant when *p* < 0.05, ^#^WT + *Hc* vs. *Il22*^−/−^ + *Hc*. *Il22*^−/−^ mice infected with lethal inoculum (1 × 10^6^ yeasts) of *H. capsulatum* were treated 1× a day with 1 μg rIFN- γ (i.n.) from the 4th day of infection. On day 7 post-infection, the medium right lobe was removed, fixed in formalin and embedded in paraffin. Five-micrometer sections were stained by HE or Grocott’s method (**G**) and analyzed by light microscopy, with a magnification of 200× and 100× (in set). Images (400× resolution) were quantified in the ImageJ software, regarding % of infiltrated area (**D**) or % of area covered by yeasts (**E**). To determine the survival rate, animals from all groups were observed daily (**H**). Data were analyzed by the log-rank test and Mantel–Cox in the post-test (*n* = 6). Data were considered statistically significant when *p* < 0.05. ^#^*Il22*^−/−^ + *Hc* vs. *Il22*^−/−^ + *Hc* + IFN-γ.

To investigate the mechanisms involved with reduced Th1 cells observed in *Il22*^−/−^ mice infected with *H. capsulatum*, we hypothesized that the deficiency of this cytokine leads to the impaired production of factors that are required for Th1 cell differentiation and IFN-γ production, such as IL-12p70 and IL-18, or related to Th17 immunity, such as IL-23. We quantified levels of IL-12p70 (Figure 6A), IL-18 (Figure 6B) and IL-23 (Figure 6C) in lung parenchyma at days two and seven post-infection. None of these cytokines were downregulated in the absence of IL-22, indicating that there is no association between reduced IFN-γ and these cytokines. Corroborating this phenomenon, the ability of IL-18 to enhance IFN-γ secretion in CD4^+^ T cells was measured ex vivo in lung cells isolated of *H. capsulatum* infected-mice. Isolated lung cells were treated with murine recombinant IL-18 (50 ng.mL^−1^) for 12 h, but the treatment did not enhance the production of IFN-γ by CD4^+^ or CD8^+^ T cells (Figure S5). As an experimental control, the production of IL-17 by CD4^+^ or CD8^+^ T cells were also evaluated, and no differences were found in IL-18-treated cells compared to untreated cells (Figure S5).

Since IFN-γ drives protective molecular mechanisms against *H. capsulatum* infection, treatment with this recombinant cytokine should revert the susceptibility of *Il22*^−/−^ mice. Indeed, the treatment of *Il22*^−/−^ mice with rIFN-γ (1 µg) reduced yeast replication in the lung parenchyma (Figure 6E,G) with no effect on lung inflammation (Figure 6D,G) observed at seven days post-infection. Notably, IFN-γ treatment reduced NO_2_^−^ in the lung parenchyma of *Il22*^−/−^ mice at the same time point after *H. capsulatum* infection (Figure 6F). Moreover, treatment of *Il22*^−/−^ mice with rIFN-γ significantly extended animal survival (Figure 5H), suggesting that IL-22 operates via IFN-γ to promote anti-*H. capsulatum* immunity and mice survival.

## 4. Discussion

IL-22 is a pleiotropic cytokine with pro and anti-inflammatory properties and plays contrasting functions in diverse inflammatory or infectious diseases. These effects depend on the inflammatory microenvironment, including the presence of other cytokines [52]. We demonstrated that *H. capsulatum* infection induces the production of IL-22 in the lungs, which was increased 7 days after infection and corroborates previous studies using conidia or/and yeast forms [38,39]. Despite that, the function of IL-22 during histoplasmosis was largely unknown. Herein, we demonstrated that this cytokine plays a critical role for resistance to *H. capsulatum* infection. These observations are in line with responses to infections by other fungal pathogens, such as *Candida albicans*. Similarly, to our observations, IL-22 was crucial for animal resistance and the control of yeast replication, while its deficiency promoted increased neutrophil infiltration into sites of infection [53,54]. Importantly, despite the deleterious role played by the accumulation of neutrophils in several conditions [37,55,56], enhanced neutrophil accumulation in the lung does not seem to be responsible for *Il22*^−/−^ mice mortality during experimental histoplasmosis.

During vulvovaginal candidiasis, IL-22-deficient mice also showed pronounced neutrophil recruitment to the vaginal epithelium, followed by increased production of AMPs, such as S100A8 and S100A9, as well as elevated tissue damage [37]. Although AMPs production has been considered an important output of IL-22 signaling, our data suggest that this is not the case during *H. capsulatum* infection. Furthermore, it is likely that AMP have no antifungal effects towards *H. capsulatum*. Interestingly, IL-22 also promotes transcription of genes related to tissue regeneration and proliferation (*Myc*, *Ccnd1*, *Rbl2* e *Cdk4*) [57,58,59] and cell survival (*Bcl2*, *Bcl2l1* and *Mcl1*) [34,57,60], which could also mediate protection during *H. capsulatum* infection.

Our data point to the significant role of IL-22 in the induction of CD4^+^ T cell-mediated immunity, particularly for the induction of optimal Th1 response and IFN-γ production. Previous studies showed that IL-22 induces Th1 signature cytokines such as IL-18 and IFN-γ during *Toxoplasma gondii* experimental infection [55,56]. In contrast, IL-22 has been shown to promote reduced infiltration of CD4^+^ IFN-γ^+^ IL-17^+^ T cells during intestinal inflammation [37]. Both IFN-γ and IL-17 play critical roles during histoplasmosis. Indeed, IL-12p35-deficient animals (unable to produce IL-12 and to mount Th1 responses) are highly susceptible to *H. capsulatum* infection [19]. Moreover, mice lacking IL-12p40 are unable to produce IL-12 and IL-23, and thus exhibit impaired Th1 and Th17 responses. These animals are even more susceptible to histoplasmosis than *Il12p35^−/−^*, and thus indicates that both cytokines cooperate to control the infection [19]. Moreover, IL-12p40 deficient mice produces less IL-22 [54], suggesting an inflammatory loop between IL-22 and Th1/Th17 response. However, IL-12p70, IL-18 and IL-23 were not involved with lower IFN-γ secretion observed in *H. capsulatum* infection of IL-22 deficient mice.

IFN-γ induces the production of NO, a major anti-fungal molecule that promotes death of *H. capsulatum* yeasts [23,51]. We observed that both transcriptional and protein expression of NOS2 were reduced in the lungs of *Il22*^−/−^ mice infected with *H. capsulatum*, thus reduced NO production must account for the susceptible phenotype of these animals. Our data were supported by studies showing that IL-22 drives NO-dependent dysplasia and DNA damage during a murine model of colitis-associated cancer [61]. In line with reduced NOS2 expression and NO_2_^−^ production, we observed that survival of *Il22*^−/−^ mice during histoplasmosis can be significantly extended by rIFN-γ treatment. Furthermore, rIFN-γ treatment reduced fungal load in the lungs of *Il22*^−/−^ mice at seven days post-infection, as well as reduced NO_2_^−^. This result is intriguing, but we hypothesize that the clearance of yeasts from lungs with IFN-γ-induced NO at early stages might downregulate NO release at later stages, possibly via a negative feedback mechanism.

## 5. Conclusions

This study suggests that IL-22 deficiency impairs Th1 response and IFN-γ production, resulting in reduced NOS2 expression, diminished the production of NO and limited control of *H. capsulatum* yeast growth. Indeed, these findings point to IL-22 as a critical biomolecule involved in the protection to *H. capsulatum* infection and these data improve the knowledge focusing on new targets for the development of effective therapy.

## Supplementary Materials

The following are available online at https://www.mdpi.com/2218-273X/10/6/865/s1, Figure S1: Gene expression of *Il22*^−/−^/WT mice during *H. capsulatum infection*, Figure S2: Lung transcriptional expression of *Defa1*, *Defb1*, *Camp*, *Lcn2* and *Muc5ac* during *H. capsulatum infection*, Figure S3: Depletion of circulating neutrophils did not enhance survival of *Il22*^−/−^ mice infected with *H. capsulatum* and Figure S4: In vitro stimulation of lung parenchyma with IL-18 did not increase polarization of Th1 or Th17 cells.

## References

[1] Antinori S. (2014). *Histoplasma capsulatum*: More widespread than previously thought. Am. J. Trop. Med. Hyg..

[2] Colombo A.L., Tobón A., Restrepo A., Telles F.D.Q., Nucci M. (2011). Epidemiology of endemic systemic fungal infections in Latin America. Med. Mycol..

[3] Horwath M.A., Fecher R., Deepe G.S. (2015). *Histoplasma capsulatum*, lung infection and immunity. Futur. Microbiol..

[4] Goodwin A.R., Shapiro J.L., Thurman G.H., Thurman S.S., Prez R.M.D. (1980). Disseminated histoplasmosis: Clinical and pathologic correlations. Md. Med. J..

[5] McKinsey D.S., Spiegel R.A., Hutwagner L., Stanford J., Driks M.R., Brewer J., Gupta M.R., Smith D.L., O’Connor M.C., Dall L. (1997). Prospective study of histoplasmosis in patients infected with human immunodeficiency virus: Incidence, risk factors, and pathophysiology. Clin. Infect. Dis..

[6] Heninger E., Hogan L.H., Karman J., Macvilay S., Hill B., Woods J.P., Sandor M. (2006). Characterization of the *Histoplasma capsulatum*-Induced Granuloma1. J. Immunol..

[7] Cain J.A., Deepe G.S. (1998). Evolution of the Primary Immune Response to *Histoplasma capsulatum* in Murine Lung. Infect. Immun..

[8] Inoue M., Shinohara M.L. (2014). Clustering of Pattern Recognition Receptors for Fungal Detection. PloS Pathog..

[9] Sorgi C.A., Secatto A., Fontanari C., Turato W.M., Belangér C., Medeiros A.I., Kashima S., Marleau S., Covas D.T., Bozza P.T. (2009). *Histoplasma capsulatum* Cell Wall β-Glucan Induces Lipid Body Formation through CD18, TLR2, and Dectin-1 Receptors: Correlation with Leukotriene B_4_ Generation and Role in HIV-1 Infection. J. Immunol..

[10] Funk C. (2001). Prostaglandins and Leukotrienes: Advances in Eicosanoid Biology. Science.

[11] Pereira P.A.T., Assis P.A., Prado M.K.B., Ramos S.G., Aronoff D.M., Paula-Silva F., Sorgi C.A., Faccioli L.H. (2017). Prostaglandins D_2_ and E_2_ have opposite effects on alveolar macrophages infected with *Histoplasma capsulatum*[S]. J. Lipid Res..

[12] Secatto A., Rodrigues L.C., Serezani C., Ramos S.G., Dias-Baruffi M., Faccioli L.H., Medeiros A.I. (2012). 5-Lipoxygenase Deficiency Impairs Innate and Adaptive Immune Responses during Fungal Infection. PLoS ONE.

[13] Prado M.K.B., Locachevic G.A., Zoccal K.F., Paula-Silva F., Fontanari C., Ferreira J.C., Pereira P.A.T., Gardinassi L.G., Ramos S.G., Sorgi C.A. (2017). Leukotriene B_4_ is essential for lung host defence and alpha-defensin-1 production during *Achromobacter xylosoxidans* infection. Sci. Rep..

[14] Medeiros A.I., Sá-Nunes A., Turato W.M., Secatto A., Frantz F.G., Sorgi C.A., Serezani C., Deepe G.S., Faccioli L.H. (2008). Leukotrienes are potent adjuvant during fungal infection: Effects on memory T cells. J. Immunol..

[15] Secatto A., Soares E.M., Locachevic G.A., Assis P.A., Paula-Silva F., Serezani C., Medeiros A.I., Faccioli L.H. (2014). The Leukotriene B_4_/BLT_1_ Axis Is a Key Determinant in Susceptibility and Resistance to Histoplasmosis. PLoS ONE.

[16] Schnizlein-Bick C., Durkin M., Kohler S., Connolly P., Lemonte A., Garringer T., Goldberg J., Smedema M., Brizendine E., Wheat L.J. (2003). Effects of CD4 and CD8 T lymphocyte depletion on the course of histoplasmosis following pulmonary challenge. Med. Mycol..

[17] Lin J.-S., Wu-Hsieh B.A.-Y. (2004). Functional T cells in primary immune response to histoplasmosis. Int. Immunol..

[18] Allendoerfer R., Deepe G.S. (1997). Intrapulmonary response to *Histoplasma capsulatum* in gamma interferon knockout mice. Infect. Immun..

[19] Deepe G.S., Gibbons R.S. (2009). Interleukins 17 and 23 influence the host response to *Histoplasma capsulatum*. J. Infect. Dis..

[20] Allendoerfer R., Deepe G.S. (2000). Regulation of infection with *Histoplasma capsulatum* by TNFR1 and -2. J. Immunol..

[21] Deepe G.S., McGuinness M. (2006). Interleukin-1 and host control of pulmonary histoplasmosis. J. Infect. Dis..

[22] Cain J.A., Deepe G.S. (2000). Interleukin-12 Neutralization Alters Lung Inflammation and Leukocyte Expression of CD80, CD86, and Major Histocompatibility Complex Class II in Mice Infected with *Histoplasma capsulatum*. Infect. Immun..

[23] Lane E.T., Otero G.C., Wu-Hsieh B.A.-Y., Howard D.H. (1994). Expression of inducible nitric oxide synthase by stimulated macrophages correlates with their antihistoplasma activity. Infect. Immun..

[24] Zhou P., Sieve M.C., Bennett J., Kwon-Chung K.J., Tewari R.P., Gazzinelli R.T., Sher A., A Seder R. (1995). IL-12 prevents mortality in mice infected with *Histoplasma capsulatum* through induction of IFN-gamma. J. Immunol..

[25] Kroetz D.N., Deepe G.S. (2011). The role of cytokines and chemokines in *Histoplasma capsulatum* infection. Cytokine.

[26] Deepe G.S., Gibbons R.S. (2008). TNF-α Antagonism Generates a Population of Antigen-Specific CD4+CD25+ T Cells That Inhibit Protective Immunity in Murine Histoplasmosis1. J. Immunol..

[27] Peng J.-K., Lin J.-S., Kung J.T., Finkelman F.D., Wu-Hsieh B.A.-Y. (2004). The combined effect of IL-4 and IL-10 suppresses the generation of, but does not change the polarity of, type-1 T cells in Histoplasma infection. Int. Immunol..

[28] Eyerich S., Eyerich K., Pennino D., Carbone T., Nasorri F., Pallotta S., Cianfarani F., Odorisio T., Traidl-Hoffmann C., Behrendt H. (2009). Th22 cells represent a distinct human T cell subset involved in epidermal immunity and remodeling. J. Clin. Investig..

[29] Hansson M., Silverpil E., Lindén A., Glader P. (2013). Interleukin-22 produced by alveolar macrophages during activation of the innate immune response. Inflamm. Res..

[30] Malhotra N., Yoon J., Castillo J.M.L., Galand C., Archer N., Miller L.S., Geha R.S. (2016). IL-22 derived from γδ T cells restricts *Staphylococcus aureus* infection of mechanically injured skin. J. Allergy Clin. Immunol..

[31] Zindl C.L., Lai J.-F., Lee Y.K., Maynard C.L., Harbour S.N., Ouyang W., Chaplin D., Weaver C.T. (2013). IL-22–producing neutrophils contribute to antimicrobial defense and restitution of colonic epithelial integrity during colitis. Proc. Natl. Acad. Sci. USA.

[32] Res P.C.M., Piskin G., De Boer O.J., Van Der Loos C.M., Teeling P., Bos J.D., Teunissen M.B.M. (2010). Overrepresentation of IL-17A and IL-22 Producing CD8 T Cells in Lesional Skin Suggests Their Involvement in the Pathogenesis of Psoriasis. PLoS ONE.

[33] Kumar P., Thakar M.S., Ouyang W., Malarkannan S. (2012). IL-22 from conventional NK cells is epithelial regenerative and inflammation protective during influenza infection. Mucosal Immunol..

[34] Sonnenberg G.F., Fouser L.A., Artis D. (2010). Functional Biology of the IL-22-IL-22R Pathway in Regulating Immunity and Inflammation at Barrier Surfaces. Adv. Immunol..

[35] Wozniak K., Hole C.R., Yano J., Fidel P.L., Wormley F.L. (2014). Characterization of IL-22 and antimicrobial peptide production in mice protected against pulmonary *Cryptococcus neoformans* infection. Microbiology.

[36] Gessner M.A., Werner J.L., Lilly L.M., Nelson M.P., Metz A.E., Dunaway C.W., Chan Y.R., Ouyang W., Brown G.D., Weaver C.T. (2011). Dectin-1-Dependent Interleukin-22 Contributes to Early Innate Lung Defense against *Aspergillus fumigatus*. Infect. Immun..

[37] Borghi M., De Luca A., Puccetti M., Jaeger M., Mencacci A., Oikonomou V., Pariano M., Garlanda C., Moretti S., Bartoli A. (2015). Pathogenic NLRP3 Inflammasome Activity during Candida Infection Is Negatively Regulated by IL-22 via Activation of NLRC4 and IL-1Ra. Cell Host Microbe.

[38] Sahaza J.H., Suárez-Alvarez R., Estrada-Bárcenas D.A., Pérez-Torres A., Taylor M.L. (2015). Profile of cytokines in the lungs of BALB/c mice after intra-nasal infection with *Histoplasma capsulatum* mycelial propagules. Comp. Immunol. Microbiol. Infect. Dis..

[39] Kroetz D.N., Deepe G.S. (2010). CCR5 Dictates the Equilibrium of Proinflammatory IL-17+and Regulatory Foxp3+T Cells in Fungal Infection. J. Immunol..

[40] Medeiros A.I., Sá-Nunes A., Soares E.G., Peres C.M., Silva C., Faccioli L.H. (2004). Blockade of Endogenous Leukotrienes Exacerbates Pulmonary Histoplasmosis. Infect. Immun..

[41] Locachevic G.A., Pereira P.A.T., Secatto A., Fontanari C., Galvão A.F., Prado M.K.B., Zoccal K.F., Petta T., De Moraes L.A.B., Ramos S.G. (2015). Erythropoietin Exacerbates Inflammation and Increases the Mortality of *Histoplasma capsulatum*-Infected Mice. Mediat. Inflamm..

[42] Yamamoto N., Shibamori M., Ogura M., Seko Y., Kikuchi M. (1998). Effects of Intranasal Administration of Recombinant Murine Interferon-γ on Murine Acute Myocarditis Caused by Encephalomyocarditis Virus. Circulation.

[43] Clemons K.V., Lutz J.E., Stevens D.A. (2001). Efficacy of Recombinant Gamma Interferon for Treatment of Systemic Cryptococcosis in SCID Mice. Antimicrob. Agents Chemother..

[44] Reed S.G. (1988). In vivo administration of recombinant IFN-gamma induces macrophage activation, and prevents acute disease, immune suppression, and death in experimental *Trypanosoma cruzi* infections. J. Immunol..

[45] Souza C.O.S., Espíndola M.S., Fontanari C., Prado M.K.B., Frantz F.G., Rodrigues V., Gardinassi L.G., Faccioli L.H. (2018). CD18 Regulates Monocyte Hematopoiesis and Promotes Resistance to Experimental Schistosomiasis. Front. Immunol..

[46] Tristão F.S.M., Rocha F.A., Carlos D., Ketelut-Carneiro N., Souza C.O.S., Milanezi C.M., Silva J.S. (2017). Th17-Inducing Cytokines IL-6 and IL-23 Are Crucial for Granuloma Formation during Experimental Paracoccidioidomycosis. Front. Immunol..

[47] Sorgi C.A., Peti A.P.F., Petta T., Meirelles A.F.G., Fontanari C., De Moraes L.A.B., Faccioli L.H. (2018). Comprehensive high-resolution multiple-reaction monitoring mass spectrometry for targeted eicosanoid assays. Sci. Data.

[48] Liang S.C., Tan X.-Y., Luxenberg D.P., Karim R., Dunussi-Joannopoulos K., Collins M., Fouser L.A. (2006). Interleukin (IL)-22 and IL-17 are coexpressed by Th17 cells and cooperatively enhance expression of antimicrobial peptides. J. Exp. Med..

[49] Wolk K., Witte E., Witte K., Warszawska K., Sabat R. (2010). Biology of interleukin-22. Semin. Immunopathol..

[50] Deepe G.S., Gibbons R.S. (2003). Protective and memory immunity to *Histoplasma capsulatum* in the absence of IL-10. J. Immunol..

[51] Nakamura L.T., Wu-Hsieh B.A.-Y., Howard D.H. (1994). Recombinant murine gamma interferon stimulates macrophages of the RAW cell line to inhibit intracellular growth of *Histoplasma capsulatum*. Infect. Immun..

[52] Rutz S., Eidenschenk C., Ouyang W. (2013). IL-22, not simply a Th17 cytokine. Immunol. Rev..

[53] De Luca A., Zelante T., D’Angelo C., Zagarella S., Fallarino F., Spreca A., Iannitti R.G., Bonifazi P., Renauld J.-C., Bistoni F. (2010). IL-22 defines a novel immune pathway of antifungal resistance. Mucosal Immunol..

[54] Conti H.R., Shen F., Nayyar N., Stocum E., Sun J.N., Lindemann M.J., Ho A.W., Hai J.H., Yu J.J., Jung J.W. (2009). Th17 cells and IL-17 receptor signaling are essential for mucosal host defense against oral candidiasis. J. Exp. Med..

[55] Pinto L.G., Talbot J., Peres R.S., Franca R.F.D.O., Ferreira S.H., Ryffel B., Alves-Filho J.C., Figueiredo F., Cunha F.Q., Cunha F.Q. (2015). Joint production of IL-22 participates in the initial phase of antigen-induced arthritis through IL-1β production. Arthritis Res..

[56] Muñoz M., Eidenschenk C., Ota N., Wong K., Lohmann U., Kühl A.A., Wang X., Manzanillo P., Li Y., Rutz S. (2015). Interleukin-22 Induces Interleukin-18 Expression from Epithelial Cells during Intestinal Infection. Immun..

[57] Zenewicz L., Yancopoulos G.D., Valenzuela D.M., Murphy A.J., Karow M., Flavell R.A. (2007). Interleukin-22 but Not Interleukin-17 Provides Protection to Hepatocytes during Acute Liver Inflammation. Immun..

[58] Pan H., Hong F., Radaeva S., Gao B. (2004). Hydrodynamic gene delivery of interleukin-22 protects the mouse liver from concanavalin A-, carbon tetrachloride-, and Fas ligand-induced injury via activation of STAT3. Cell. Mol. Immunol..

[59] Radaeva S., Sun R., Pan H.-N., Hong F., Gao B. (2004). Interleukin 22 (IL-22) plays a protective role in T cell-mediated murine hepatitis: IL-22 is a survival factor for hepatocytes via STAT3 activation. Hepatology.

[60] Sonnenberg G.F., Nair M.G., Kirn T.J., Zaph C., Fouser L.A., Artis D. (2010). Pathological versus protective functions of IL-22 in airway inflammation are regulated by IL-17A. J. Exp. Med..

[61] Wang C., Gong G., Sheh A., Muthupalani S., Bryant E.M., Puglisi A.D., Holcombe H., Conaway A.E., Parry N., Bakthavatchalu V. (2017). Interleukin-22 drives nitric oxide-dependent DNA damage and dysplasia in a murine model of colitis-associated cancer. Mucosal Immunol..

