# Supplementary material for: IL-22 Promotes IFN-γ-Mediated Immunity against Histoplasma capsulatum Infection
Source: Biomolecules. 2020 Jun 5;10(6):865. doi: 10.3390/biom10060865 (PMC7356283; doi:10.3390/biom10060865)
Supplement: Supplementary file 1 [file biomolecules-10-00865-s001.pdf]

Supplementary Material

IL-22 promotes IFN-γ-mediated immunity against *Histoplasma capsulatum* infection

Morgana K. B. Prado [1,2,†], Caroline Fontanari [1], Camila O. S. Souza [1,2], Luiz G. Gardinassi [1,†], Karina F. Zoccal [1,‡], Francisco W. G. de Paula-Silva [1], Ana P. F. Peti [1], Carlos A. Sorgi [1], Alyne F. G. Meirelles [1], Simone G. Ramos [3], José C. Alves-Filho [4] and Lúcia H. Faccioli [1,*]

Supplementary Material

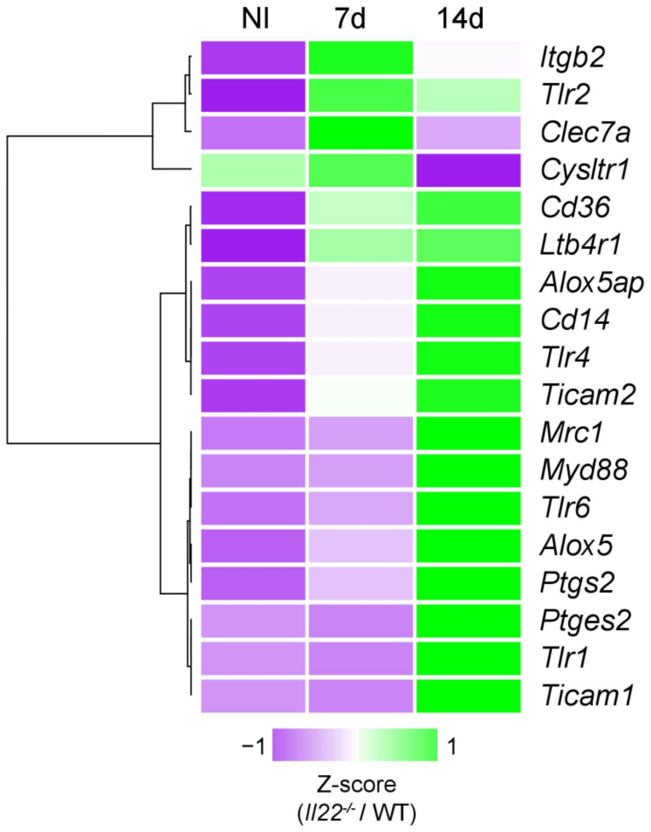

Figure S1. Gene expression of *Il22*$^{-/-}$/WT mice during *H. capsulatum infection*. A 96-well precoated plate (Mouse Custom Plates RT² Profiler PCR Array; Qiagen – Beverly, MA, EUA) together with the RT² SYBR Green qPCR master mixes (330523; Qiagen – Beverly, MA, EUA) was used to examine the relative expression set of of *Itgb2*, *Tlr2*, *Clec7a*, *Cysltr1*, *Cd36*, *Ltb4r1*, *Alox5ap*, *Cd14*, *Tlr4*, *Ticam2*, *Mrc1*, *Myd88*, *Tlr6*, *Alox5*, *Ptgs2*, *Ptges2*, *Tlr1* and *Ticam* genes in lung samples from eight-weeks-old *Il22*$^{-/-}$ and WT mice infected with *H. capsulatum* and respective controls (Eppendorf Mastercycler ep Realplex – Hamburg, Alemanha). The cycle thresholds (Ct) were used to calculate the relative expression of the target genes, which were normalized to the housekeeping gene *Tbp* (TATA-box-binding protein) and analyzed with the $2^{-\Delta\Delta Ct}$ method. The heat map was generated using the R Language and Environment for Statistical Computing v.3.5.0 and the package *gplots*. Hierarchical clustering was performed with the package *amap* using the correlation method and ward linkage. NI= non-infected; 7d and 14d = 7th and 14th days post-infection.

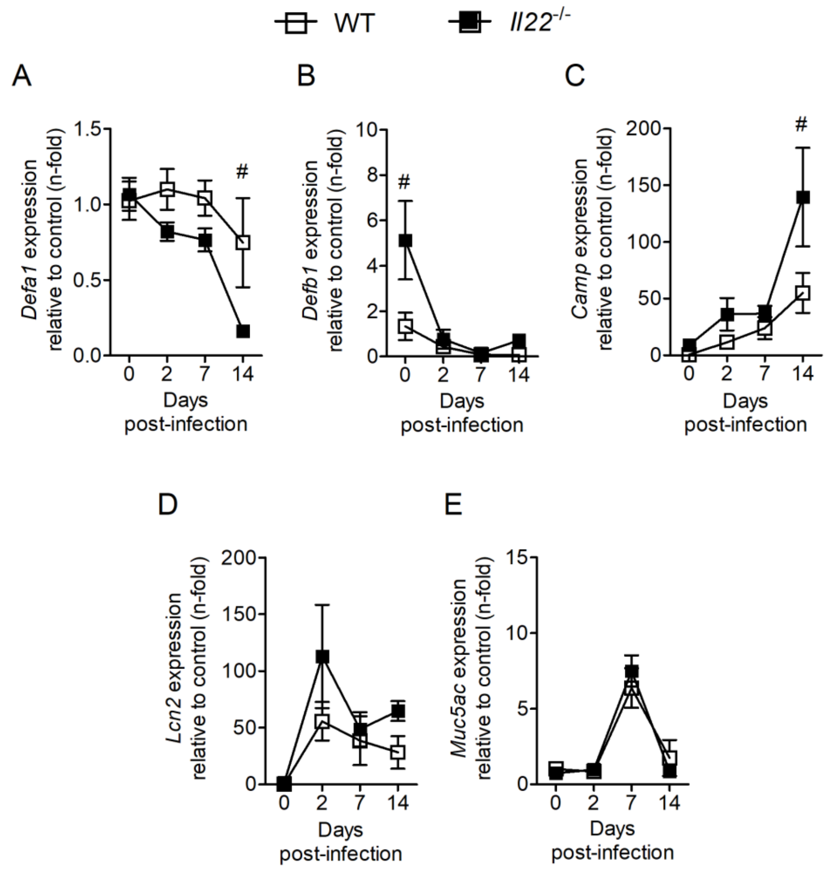

Figure S2. Lung transcriptional expression of *Defa1*, *Defb1*, *Camp*, *Lcn2* and *Muc5ac* during *H. capsulatum* infection. Eight-weeks-old WT or *Il22*$^{-/-}$ mice were infected i.t. with lethal inoculum (1 × 10^6 yeast) of *H. capsulatum*. The left lower lobe was removed and the RNA extracted for analysis of the gene expression of *Defa1* (**A**), *Defb1* (**B**), *Camp* (**C**), *Lcn2* (**D**) and *Muc5ac* (**E**) by RT qPCR using Taqman primers (Mm02524428_g1; Mm00432803_m1; Mm00438285_m1; Mm01324470_m1 and Mm01276718_m1). *Gapdh* (Mm99999915_g1) was used as reference genes and to normalize expression levels by $^{\Delta\Delta}$Ct method. The expression data was presented as n-fold difference relative to the control group. Data were expressed as mean ± SEM of a representative experiment (*n* = 4–7) and analyzed by the Two-Way ANOVA test and Bonferroni post-test. Data were considered statistically significant when $p < 0.05$, and #WT + *Hc vs. Il22*$^{-/-}$ + *Hc*.

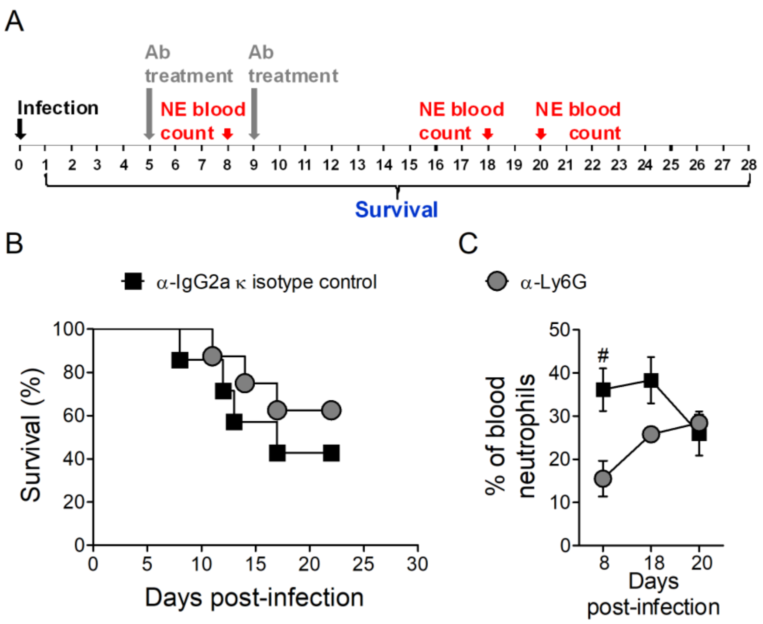

Figure S3. Depletion of circulating neutrophils did not enhance survival of *Il22*$^{-/-}$ mice infected with *H. capsulatum*. Schematic design of the neutrophil depletion experiment. Eight-weeks-old *Il22*$^{-/-}$ mice were infected with lethal inoculum (1×10^6 yeasts) of *H. capsulatum* and treated at days five and nine post-infection with 120 µg of specific-Ab (i.p.) (**A**). The animals were observed daily to determine the survival curve (**B**). Data were analyzed by Log-rank test and Mantel-Cox in the post-test (*n* = 7–8). Data were considered statistically significant when $p < 0.05$, $^{\#}$*Il22*$^{-/-}$ + *Hc* + α-Ly6G *vs.* *Il22*$^{-/-}$ + *Hc* + α-IgG2a κ isotype control. At 8th, 18th and 20th days post infection a sample of blood from tail vein were used for differential count of circulating cells using panoptic stain (**C**). Data were expressed as mean ± SEM of a representative experiment (*n* = 4–8) and analyzed by the Two-Way ANOVA test and Bonferroni post-test. Data were considered statistically significant when $p < 0.05$, and $^{\#}$*Il22*$^{-/-}$ + *Hc* + α-Ly6G *vs.* *Il22*$^{-/-}$ + *Hc* + α-IgG2a κ isotype control.

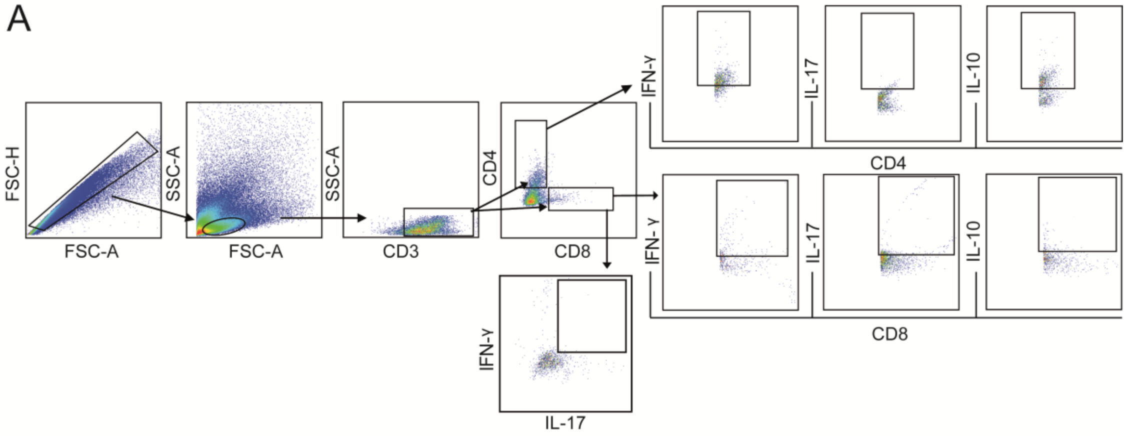

Figure S4. Schematic illustration of the gating hierarchy used for lymphocyte analysis. Pseudocolor plots show representative flow cytometric data of CD4 and CD8 T lymphocytes intracellularly stained for IFN-γ, IL-17A or IL-10 production.

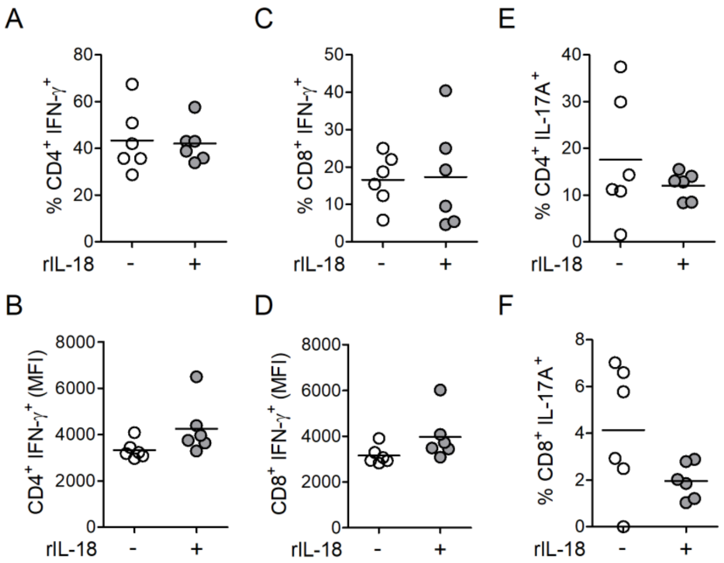

Figure S5. In vitro stimulation of lung parenchyma with IL-18 did not increase polarization of Th1 or Th17 cells. Eight-weeks-old WT or *Il22*$^{-/-}$ mice were infected i.t. with lethal inoculum (1 × 10^6 yeast) of *H. capsulatum*. The upper left lobe was removed, disrupted and stimulated or not with 50 ng.mL^{-1} of recombinant mouse IL-18, PMA, inonomycin and brefeldin A for 12 hours. The cells were then evaluated for the percentage of Th1 ($CD3^+CD4^+$IFN-γ^+) (**A**), Tc1 ($CD3^+CD8^+$IFN-γ^+) (**C**), Th17 ($CD3^+CD4^+$IL-$17A^+$) and Tc17 ($CD3^+CD8^+$IL-$17A^+$) by flow cytometry. The median of fluorescence intensity (MFI) of IFN-γ^+ $CD4^+$ (**B**) or $CD8^+$ (**D**) T cells were either evaluated. Data were analyzed by the Two-Way ANOVA test and Bonferroni in the post-test and considered significant when $p < 0.05$, #WT + *Hc vs.* WT + *Hc* + rIL-18 or #*Il22*$^{-/-}$ + *Hc vs. Il22*$^{-/-}$ + *Hc* + rIL-18 (n = 4–6) from a representative experiment.
